# Alternative Polyadenylation in response to temperature stress contributes to gene regulation in *Populus trichocarpa*

**DOI:** 10.1186/s12864-020-07353-9

**Published:** 2021-01-14

**Authors:** Chao Yan, Yupeng Wang, Tao Lyu, Zhikang Hu, Ning Ye, Weixin Liu, Jiyuan Li, Xiaohua Yao, Hengfu Yin

**Affiliations:** 1 State Key Laboratory of Tree Genetics and Breeding, Research Institute of Subtropical Forestry, Chinese Academy of Forestry, Hangzhou, 311400 Zhejiang China; 2 College of Information Science and Technology, Nanjing Forestry University, Nanjing, China; 3 Key Laboratory of Forest Genetics and Breeding, Research Institute of Subtropical, Forestry, Chinese Academy of Forestry, Hangzhou, 311400 Zhejiang China; 4 Experimental Center for Subtropical Forestry, Chinese Academy of Forestry, Fenyi, 336600 Jiangxi China

**Keywords:** Alternative polyadenylation, Abiotic stress, Temperature, *Populus*

## Abstract

**Background:**

Genome-wide change of polyadenylation (polyA) sites (also known as alternative polyadenylation, APA) is emerging as an important strategy of gene regulation in response to stress in plants. But little is known in woody perennials that are persistently dealing with multiple abiotic stresses.

**Results:**

Here, we performed a genome-wide profiling of polyadenylation sites under heat and cold treatments in *Populus trichocarpa*. Through a comprehensive analysis of polyA tail sequences, we identified 25,919 polyA-site clusters (PACs), and revealed 3429 and 3139 genes shifted polyA sites under heat and cold stresses respectively. We found that a small proportion of genes possessed APA that affected the open reading frames; and some shifts were commonly identified. Functional analysis of genes displaying shifted polyA tails suggested that pathways related to RNA metabolism were linked to regulate the APA events under both heat and cold stresses. Interestingly, we found that the heat stress induced a significantly more antisense PACs comparing to cold and control conditions. Furthermore, we showed that a unique cis-element (AAAAAA) was predominately enriched downstream of PACs in *P. trichocarpa* genes; and this sequence signal was only absent in shifted PACs under the heat condition, indicating a distinct APA mechanism responsive to heat tolerance.

**Conclusions:**

This work provides a comprehensive picture of global polyadenylation patterns in response to temperatures stresses in trees. We show that the frequent change of polyA tail is a potential mechanism of gene regulation responsive to stress, which are associated with distinctive sequence signatures.

**Supplementary Information:**

The online version contains supplementary material available at 10.1186/s12864-020-07353-9.

## Background

Alternative polyadenylation (APA) is a common post-transcriptional process of eukaryotic mRNA maturation, which generates altered 3′ ends of transcripts. The use of different polyadenylation site (polyA) has been found to play essential roles of increasing transcriptome diversity and regulating gene expression profiles [1, 2]. With the support of high throughput sequencing technologies, designated RNA sequencing approaches, such as 3′READS+ [3], PolyAdenylation Site Sequencing (PAS-Seq) [4] and Poly(A)-ClickSeq [5], that target the polyA tails of mRNA, have been developed for accurate profiling of APA.

In plants, APA is widespread and has been extensively investigated. Genome-wide identification of APA has been reported in a number of plant species, including Arabidopsis [6, 7], rice [8], bamboo [9], and more. A recent constructed database (PlantAPAdb) has recorded the APA information from six plant species for evolutionary and mechanistic analyses [10]. These studies have revealed an abundant occurrence of APA and suggested important regulatory functions at the molecular level in plants. It is clear that APA is an essential layer of post-transcriptional gene regulation involved in plant growth, development and response to endogenous and environmental signals. Generally, APA sites locate in the 3′ region of transcripts. APA sites from the 3′ untranslated regions (3’UTR) can affect the mRNA stability, translation, cellular localizations to regulate gene functions [1, 11]; APA also can be inside an open reading frame (ORF) and thereby regulate the protein function [11]; in addition, studies of the *Flowering inhibitor Locus C* (*FLC*) have shown that usage of polyA site in the antisense transcript is required for determining flowering transition in Arabidopsis and other plant species [12, 13]. Although a great number of plant APA sites is identified, the regulatory mechanism of APA associated gene expression still is poorly understood.

In recent years, the APA mediated gene regulation is found to play an important role in stress responses. Notably, abiotic stresses can induce APA events to generate distinct transcript isoforms in diverse plant species [14–16]. For example, in Arabidopsis, hypoxia has been found to induce the usage of non-canonical (e.g. 5’UTR and protein-coding) polyA sites, which suggested a negative role of gene regulation [15]. In addition, genome-wide profiling of APA in sorghum has shown that some stress-induced isoforms were associated with a unique intronic polyA signal [17]. These results indicate that global shift of polyA site in response of stress is a strategy of survival which affect a wide-range of gene functions.

*Populus trichocarpa* is a model tree species with comprehensive genome information as well as significant ecological and commercial importance [18]; and as a perennial plant, the growth and development of *P. trichocarpa* face multiple levels of abiotic stresses. Small-scale characterization of APA sites targeting 14 NAC (NAM, ATAF1/2, CUC2) genes has been reported in *P. trichocarpa*, and three APA transcripts have been found differentially expressed during hormone treatments [19]. Furthermore, functional characterization of APA isoforms of *Secondary Wall-Associated NAC Domain 1* (*SND1*) has shown that truncated SND1 can negatively regulate the function of full SND1 [20]. It is conceivable that APA-mediated gene regulation is a key aspect for development and stress response in *P. trichocarpa*. However, genome-wide profiling and analysis of APA in *P. trichocarpa* are lacking. Here, we report the genome-wide identification of APA profiles in responsive to heat and cold stresses using the PAS-seq platform in *P. trichocarpa*. We show that the change of polyA tails is widespread under the temperature stresses, and genes related to RNA metabolism are linked to APA-mediated post-transcriptional regulation. We also provide molecular evidence of unique sequence signatures associated with stress-related shifts of polyA sites in *P. trichocarpa*.

## Result

### The processing and mapping of PAS-Seq reads for identification of polyA-tail associated sequences

**Fig. 1:**
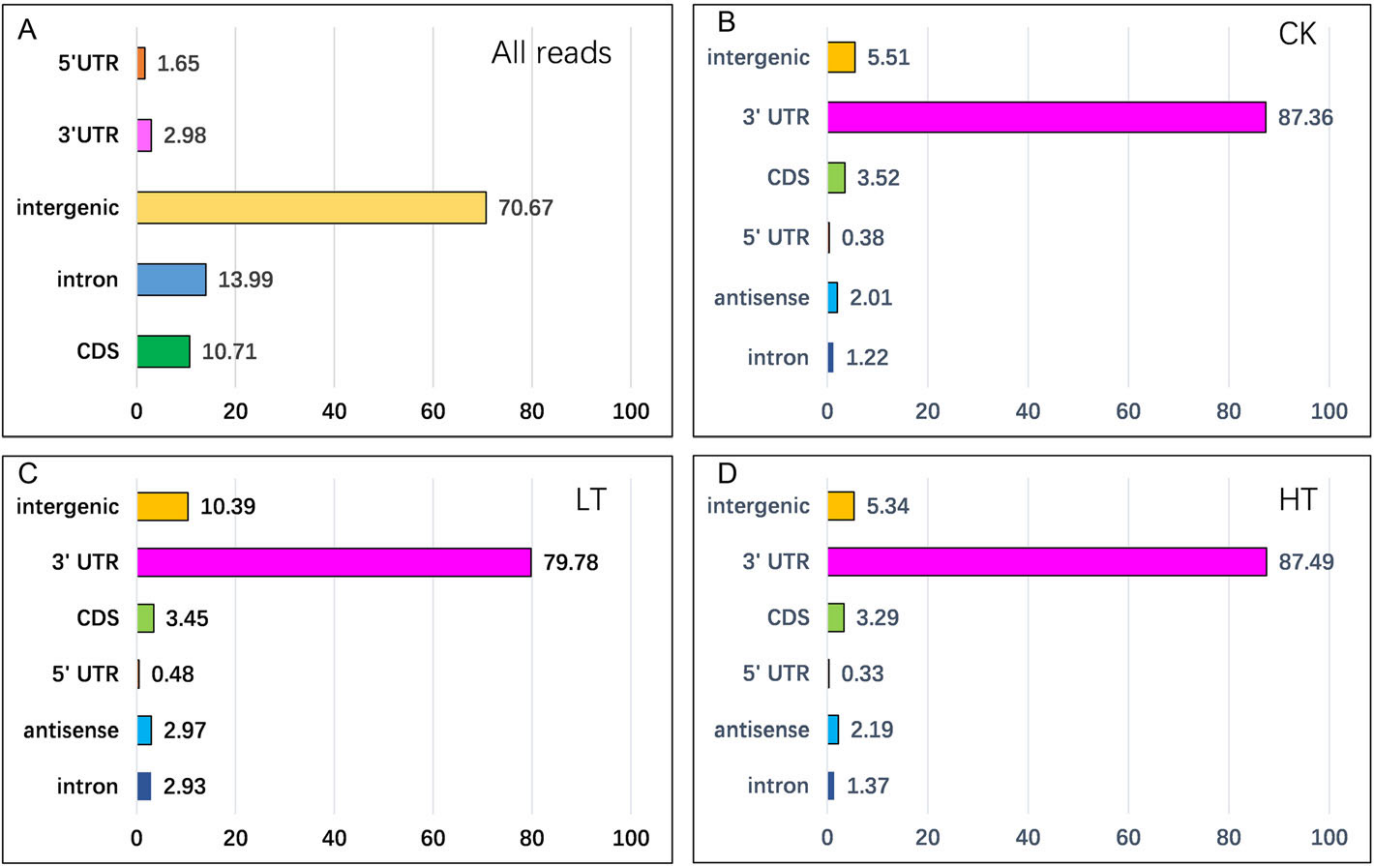
Distribution of mapped reads in the genomic regions of *P. populus.* **a** The PAS-seq reads were mapped to the reference genome. The mapped reads were categorized according to the genome annotation information. **b**-**d**, indicate mapping results of each sample using the final PAS-seq reads that pre-filtered with polyA tail information: B, control condition (CK), cold (LT), heat (HT)

To study the alternative polyadenylation pattern in *P. trichocarpa*, we performed a high-throughput transcriptome sequencing targeting the polyadenylation sites by the PAS-Seq approach. The polyA enriched libraries were constructed under different temperature stresses (4 °C and 40 °C), and the normal growth condition (24 °C) was set as the control. We sequenced the libraries using the Illumina HiSeq X Ten platform to generate 150 nt pair-end sequencing reads, and obtained 36–43 million (Supple. Table S1) raw reads for each library. The clean reads of all samples were combined and mapped to the *P. populus* genome sequences. We found that, based on the annotated genomic regions, 2.98% of total reads were located in the 3′ gene untranslated region (3’UTR) (Fig. 1a), and the majority of reads were mapped to the intergenic region (70.67%, Fig. 1a).

To retrieve the sequences upstream of the polyA tail, we first anchored the region containing at least 8 consecutive As, and then went through upstream for regions with at least 2 non-A and clipped the sequences of polyA tail; and the remaining reads with more than 11 nt in length were used for polyA signal analysis (Supple. Table S2). The PAS-seq reads were further filtered with the polyA sequences which resulted in the “final PAS-reads” of enriched with polyA tails for each sample (Supple. Table S2). Then, the “final PAS-reads” were re-mapped to the reference genome and the both strands of transcripts sequences. We found that the majority of “final PAS-reads” were from the 3’UTR region (Fig. 1b-d); and about 10% of reads were located in the intergenic areas in all samples (Fig. 1b-d); and small proportions of the reads were found in CDS, 5’UTR, antisense, and intronic regions (Fig. 1b-d). These results were agreeable with our experimental designs, suggesting that the pipeline of reads processing was credible to downstream analyses of polyA tails.

### Global characterizations of polyA tails based on the PAS-Seq sequences

**Fig. 2:**
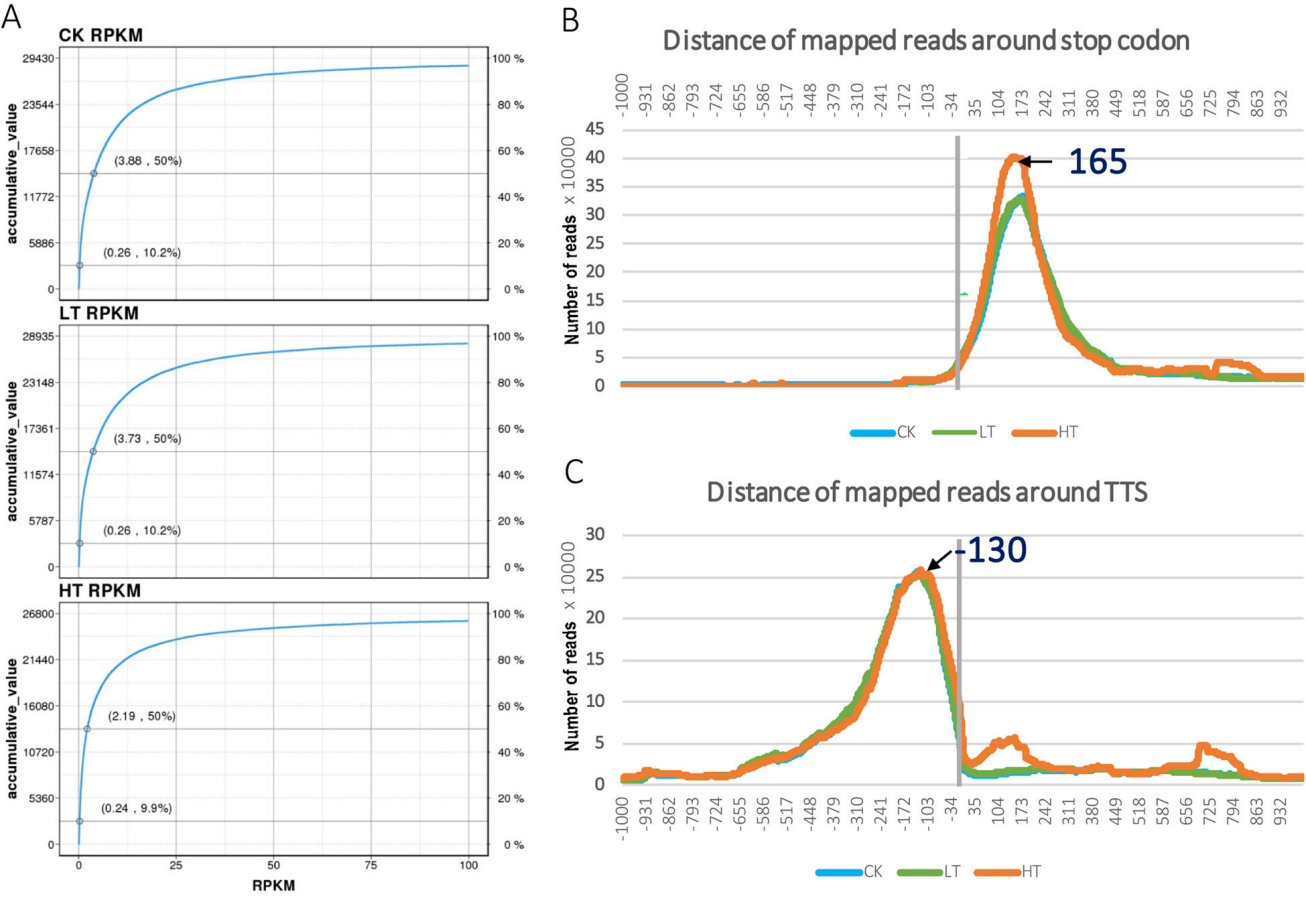
Abundance and distribution of mapped reads along gene structure. **a** the mapped reads are calculated to RPKM to reveal gene abundance, and the accumulative expression of gene levels in each sample is plotted. **b** the distribution of mapped reads 1 kb up and downstream of stop codon is plotted. C. the distribution of mapped reads 1 kb up and downstream of transcription terminal site is plotted. The arrows indicate the peaks of reads accumulation, and the associated numbers indicate the location according to the stop codon (**b**) and transcription terminal site (**c**). The vertical lines indicate the location of stop codon (**b**) and transcription terminal site (**c**)

To reveal the abundance of expressed genes, we used the “final PAS-reads” to evaluate the gene expression levels and found that 25,068 genes out of 41,335 had a higher RPKM (Reads per kilo base of a gene per million reads) value than 1. The cumulative expression levels were plotted for each library, and the average RPKM value of 50% was between 2 and 4 in different samples (Fig. 2a). To investigate the distribution pattern of reads along a transcript, we assessed the location of reads based on the annotation of gene model. We showed that the peak of reads distribution was evident at the 3′ region of transcripts (Fig. 2); when assessing the 1 kb upstream and downstream areas of TTS and stop codon, we found that the peak of reads were located closely upstream of TTS and downstream of stop codon (Fig. 2b-c). These results implied that the processed reads were efficient to determine the 3′-ends, including 3’UTR sequences and the polyA signals.

### Changes of PACs between different temperature stress conditions

**Fig. 3:**
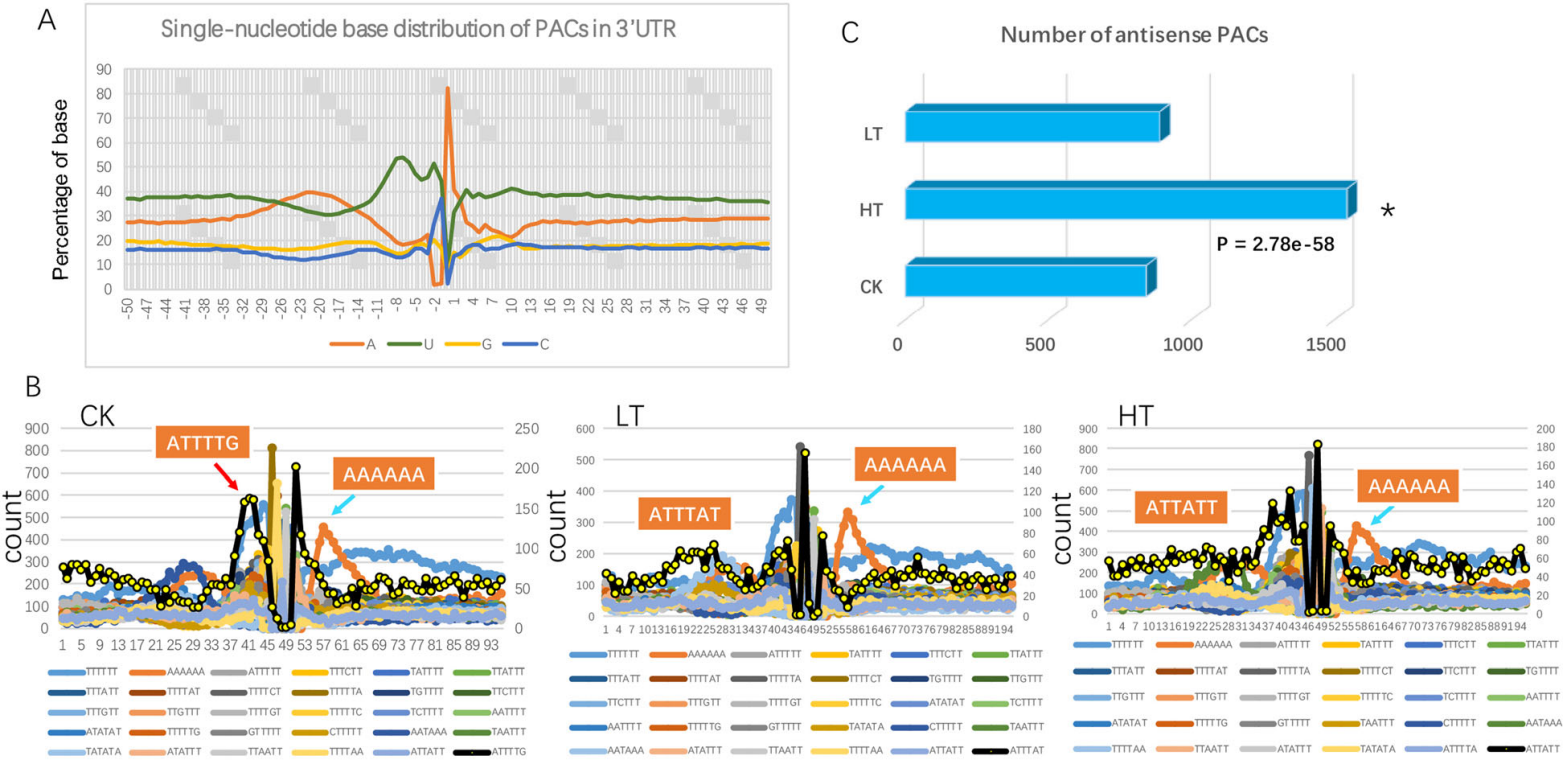
Global pattern of 3’UTR and polyA processing signals. **a** the nucleotide composition near the polyA site at the single-base resolution. **b** the distribution of top hexamers 50 bp up- and down- stream of polyA site cluster under control (CK), cold (LT) and heat (HT) conditions. Blue arrows indicate the accumulation peak of “AAAAAA” downstream of PACs in all samples. Red arrow indicates the G-containing hexamer “ATTTTG” is missed under heat and cold stresses. Left y-axis indicates the counts of hexamers, and the right y-axis indicate the highlighted hexamer in yellow with black line. Yellow color highlights unique hexamer in samples: the “ATTTG” hexamer in CK, “ATTTAT” in LT, “ATTATT” in HT. **c** The identification of antisense PACs under control, cold and heat conditions. The star indicates a significant increase of antisense PACs under heat condition thais calculated by the right-sided Fisher’s extract test

The polyA containing reads were subjected to clustering analysis to identify the polyA-site cluster (PAC) based on the location and relative abundance of PAS-reads. The samples of different treatments were independently analyzed for PAC identification, and combined reads were also used to reveal the total PAC sites. We showed that the PAC sites were dominantly located in the 3’UTR regions of genes (Supple. Table S3). The nucleotide composition pattern around PAC sequences were studied. The 50 bp upstream and downstream regions were scanned to display the nucleotide composition using combined reads (Fig. 3a). We revealed that the regions were dominant with A and U, and A and U showed distinct but complementary distribution patterns (Fig. 3a). A spike of A was revealed at the polyA site indicating the cleavage region; and a spike of C was also found just in front of the A spike (Fig. 3a). The profile of the nucleotides was consistent with polyA sites in other plant species suggesting a conserved mechanism of polyadenylation.

To determine the signal elements around PAC sequences, the 50 bp upstream and downstream regions were examined to identify the patterns of sequences. The 6-nt window of nucleotides was used to reveal the common patterns (Loke et al. 2005). The top 30 signals were displayed, and we found that “AATAAA” and “ATAAAA” were most frequent in all samples except for the all “T” and all “A” hexamers; and the locations of “AATAAA” and “ATAAAA” were consistent between samples around the 16–26 nt upstream area (Fig. 3b), supporting that those were canonical polyA signals. Interestingly, we showed that there was one unique hexamer that was specific to sample: “ATTTTG” in control, “ATTTTTA” in cold stress, “TTTTAA” in heat stress (Fig. 3b); and both heat and cold samples missed the G-containing hexamer close to the polyA cleavage site (Fig. 3b). These results suggest that the temperature stress may induce the usage of different polyA signals to generate distinctive alternative splicing isoforms. We also identified a conspicuous enrichment of “AAAAAA” 5–10 downstream the polyA cleavage site in all samples (Fig. 3b), supporting a new APA sequence signature in *P. trichocarpa*. Furthermore, we noticed that a small fraction of PACs was derived from antisense transcripts (Supple. Table S3). To further investigate the occurrence of antisense PACs between samples, we quantified the PACs from antisense, and showed that the heat condition extensively promoted the generation of antisense PACs (near 2 fold increase), but not in cold condition (Fig. 3c).

### The characterization of sequence signatures of shifted PACs between temperature stresses

**Fig. 4:**
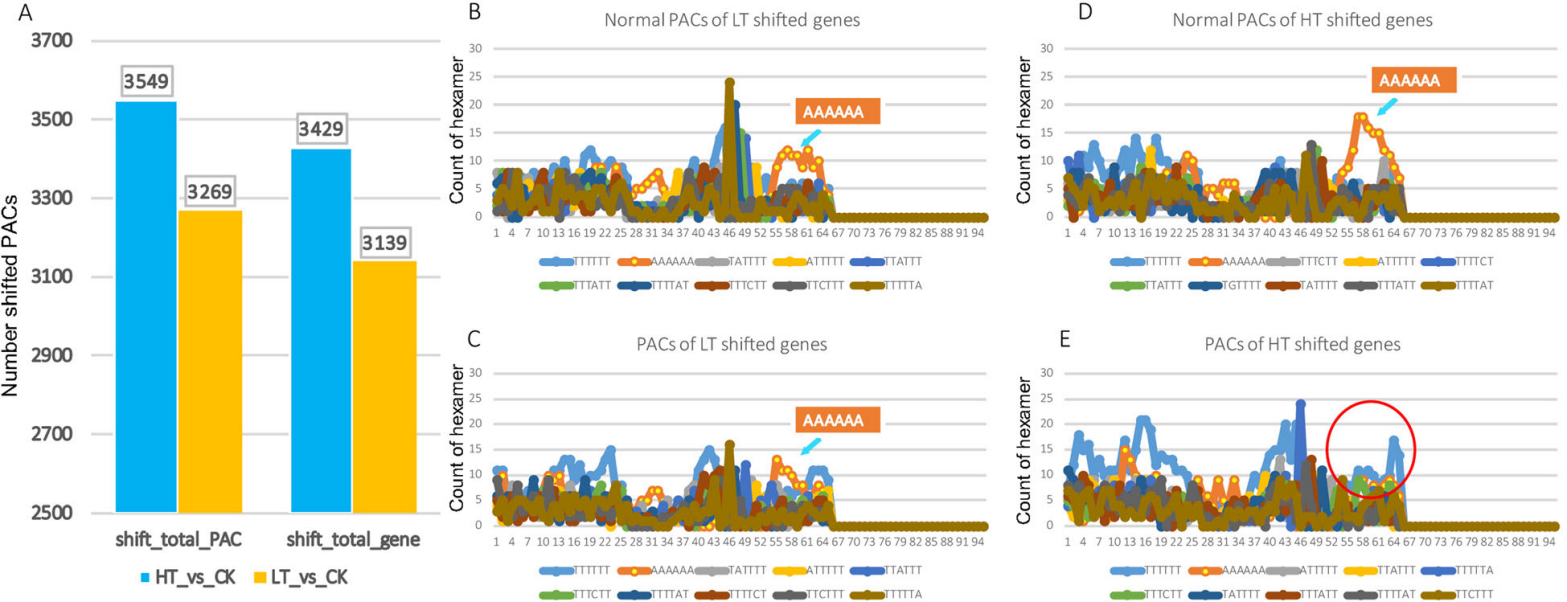
Global shifts of non-canonical PACs under cold and heat stress conditions. **a** the distribution of numbers of shifted PACs and located genes under cold and heat conditions. **b**-**c** the display of hexamers 50 bp up- and down- stream of PACs of shifted genes under the cold condition (LT). **d**-**e** the display of hexamers 50 bp up- and down- stream of PACs of shifted genes under the heat condition (HT). The blue arrows indicate the canonical enrichment of “AAAAAA” downstream of PACs that are absent under the heat-induced shifted PACs

The genes with multiple PACs (proximal and distal PACs) were analyzed to uncover APA patterns in response to temperature stresses. We found that there were 3429 and 3139 genes that possessed the shifts of PACs under heat and cold stresses respectively (Supple. Dataset 2, 3; Fig. 4a). To investigate the signals for usage of shifted PACs, the sequences near PACs in cold and heat conditions were analyzed to compare with the PACs under control condition (Fig. 4b-e). We analyzed the frequencies of hexamers in the 50 upstream and downstream regions, and found that “TTTTTT” and “AAAAAA” were most abundant in all samples, which was consistent with the previous result (Fig. 4b-e, Fig. 3b). Notably, the enrichment of “AAAAAA” at the downstream of polyA tail cleavage site was missing under heat stress condition, while it remained detectable under cold stress (Fig. 4e). It suggested that the heat stress could have promoted the selection of non-canonical polyA sites that are compromised under normal conditions.

### Characterizations of genes with shifted PACs under cold and heat stresses

**Fig. 5:**
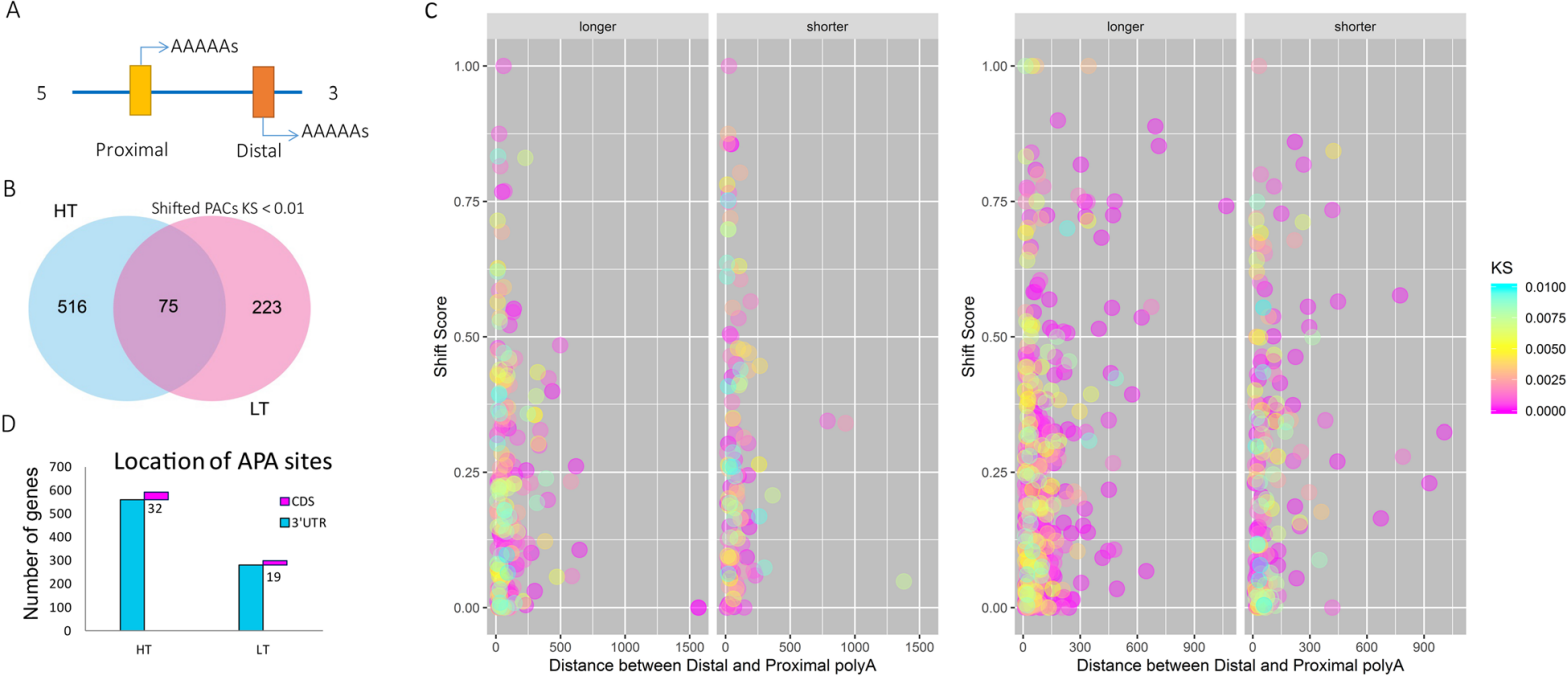
Global shifts of non-canonical PACs under cold and heat stress conditions. **a** a schematic display of distal and proximal polyA tails. **b** the venn diagram of shifted genes under heat (HT) and cold (LT) conditions (Kolmogorov-Smirnov test, *p*-Value cutoff 0.01). **c** the distribution of distance between shifted polyA tails. Left panel, cold stress; Right panel, heat stress. **d** distribution of APA in 3’UTR and coding regions (CDS). Only 32 and 19 events were found in the CDS regions under LT and HT respectively. Blue, 3’UTR PACs; Magenta, CDS PACs

To understand the temperature stress induced polyA sites, genes with shifted distal and proximal PACs were investigated (Fig. 5). We focused on the genes that were significantly responsive to treatments (Kolmogorov-Smirnov test, *p*-Value< 0.01). We found that only a small proportion of genes both changed the polyA tails under heat and cold, indicating a distinctive responsive mechanism of APA under heat and cold conditions (Fig. 5b); and majority of the change of 3′ end was within 250 bp, but the heat condition tended to generate more longer or shorter ends (Fig. 5d-e). We further assessed the potential consequences of the gene coding region, and uncovered that there were 32 and 19 genes with altered CDS under heat and cold conditions respectively, due to the APA events (Fig. 5c).

**Fig. 6:**
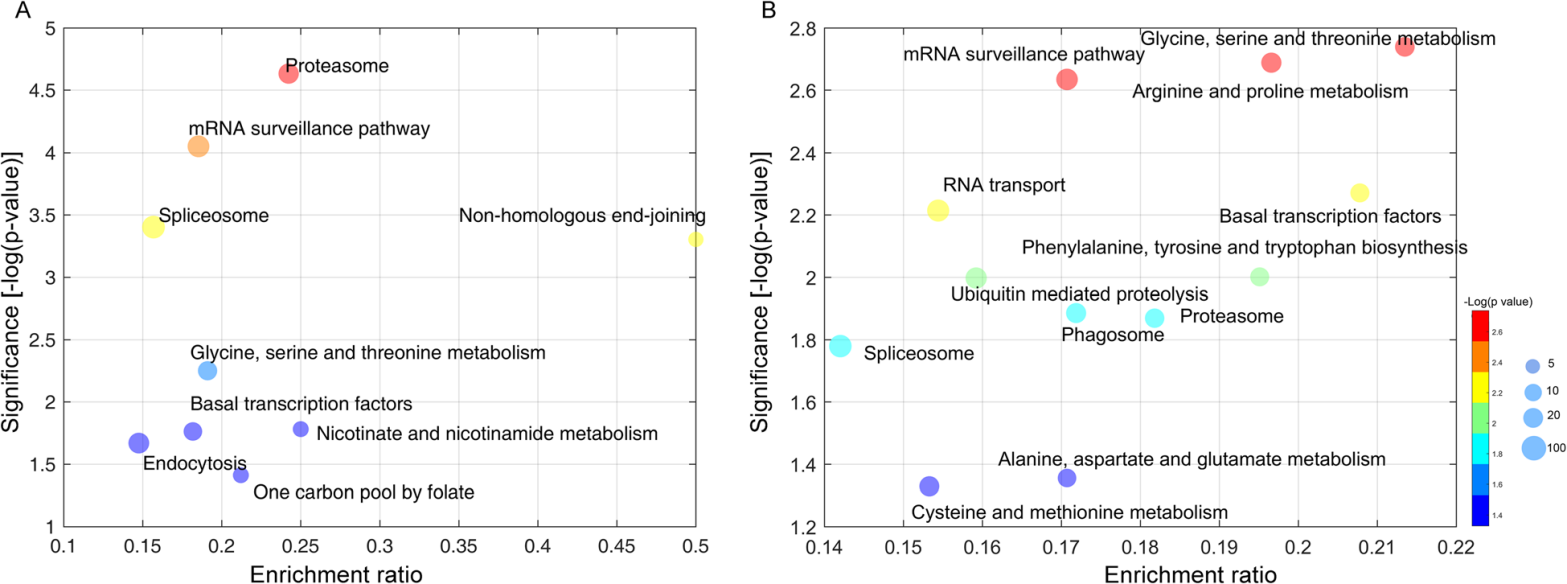
The functional enrichment of genes undergone APA under heat and cold stresses. **a** the enriched KEGG pathways under cold stress. **b** the enriched KEGG pathways under heat stress. The red arrows indicate the pathways that are both enriched under cold and heat stresses. The size of dots is correlated with number of genes that are annotated in each functional category. X-axis indicates the enrichment ratio of each functional category, which is calculated by Number genes/All genes; Y-axis indicates the –log (corrected *p*-value)

To investigate the function of genes with shifted PACs, GO enrichment analysis was performed to identify the over-representative functional GO terms (Supple. Dataset 6). We found that heat and cold stresses enriched several GO terms involved in the RNA metabolism, such as “RNA splicing”, “RNA processing” and “RNA binding” (Supple. Dataset 6). And GO terms related to direct gene expression regulation, including “regulation of transcription”, “regulation of transcription factor” and “transcription by RNA polymerase II”, were revealed under the heat condition (Supple. Dataset 6). We further analyzed the functions of shifted genes through KEGG enrichment analysis. We showed that 9 and 12 KEGG pathways were significantly enriched under the cold and heat stresses respectively (Fig. 6). Some important pathways related to gene transcription and regulation, including “Spliceosome”, “mRNA surveillance pathway”, “Basal transcription factors”, were both enriched under cold and heat (Fig. 6). Taken together, these results suggested that the cold and heat stresses induced a significant change of the polyadenylation process, which might contribute to the stress responses.

## Discussion

The survival of plants under some extreme stress conditions (e.g. heat, cold, salt, drought, and etc.) is dependent on the coordinated responses of various biochemical and physiological processes that are controlled by changes of gene expression. In recent years, global change of polyA site has been found to play an important role of gene regulation in plants [21]. Particularly, non-canonical transcript isoforms that are induced by APA under stress conditions are emerging as a transcriptomic signature to understand the transcriptional and post-transcriptional regulation of gene expression [15].

*P. trichocarpa*, as a model tree species, is an ecologically and economically important woody plant all over the world. With the wide-span distribution of environmental conditions, *P. trichocarpa* withstands multiple stresses to survive under natural environments [22]. In *P. alba*, a transcriptomic study using single-molecule long-read sequencing technology has revealed 10,213 APA sites including 2212 genes with more than one polyadenylation sites [23]. We have shown here that a widespread APA under the cold and heat stresses in *P. trichocarpa*. A total of 25,919 PACs (TPM > 5) have been identified covering over 20,000 genes (Supple. Table S3). We have revealed that there are 3429 and 3139 genes that shifted the polyA sites upon heat and cold stresses respectively (Fig. 4a). These results indicate that the change of polyA tail is a common responsive phenomenon to stress in *P. trichocarpa*. In this work, we find that around 60–70% of PACs locate in the 3’UTR representing the canonical polyA sites (Supple. Table S3). For those non-canonical PACs (e.g. 5’UTR, CDS, antisense), a complementary characterization focusing on stress-induced isoforms will be valuable to reveal potential roles of APA-mediated gene regulation. One striking difference between cold and heat is the induction of antisense PACs. The heat stress induced about 2 fold antisense PACs comparing to cold and control conditions. The role of APA-mediated antisense transcripts has been extensively investigated at the FLC locus in Arabidopsis [24]. The subset of FLC antisense transcripts are regulated by various developmental and environmental cues to regulate flowering transition [25]. Our results of antisense PACs imply that specific APA events responsive to heat stress could be involved in generating regulatory antisense transcripts to regulate gene functions.

Through sequence analysis, we show that the signatures associated with polyA tails are conserved with other plant species (Fig. 3a, b); the hexamers, such as “TTTTTT”, “AAAAAA”, “AATAAA”, “ATATAT” and etc., are commonly enriched in the near upstream element regions (NUE) [10]. Also, the signatures remain largely identical between control and stress conditions in *P. trichocarpa* (Fig. 3b). One exception is that: the G-containing hexamer “ATTTTG” in NUE is missing in heat and cold stresses (Fig. 3b). It suggests that the change of polyA tails under stresses may require the alteration of designated sequence contexts. A comparative study between sorghum and maize, two closely related monocots, has shown that preference of sequence signatures associated with PACs were influenced by species and tissue types [26]. A recent study from *Camellia japonica* has revealed that certain hexamers are preferentially selected for the polyadenylation of coding and non-coding transcripts [27]. Taken these together, our results provide insights of extensive APA events in generating transcriptome diversity and gene regulation responsive to temperature stresses. One disadvantage of PAS-seq is that it doesn’t allow for identifying complementary transcript isoforms. Therefore, a direct isoform sequencing would be valuable for investigating the APA-mediated transcripts in future.

The APA sequence signals around the cleavage site has been identified as cleavage elements (CE) in Arabidopsis [28]. We find that, in the genes displayed the shifting of polyA tails under heat and cold stress, the sequence signatures are changed considerably (Fig. 4b-e). Particularly, we have shown that the enrichment of “AAAAAA” downstream the polyA cleavage site was missed under the heat condition (Fig. 4e). In Arabidopsis, the “AAAAAA” is not identified from the comparable region, and CE is usually rich in Ts [28]. Interestingly, in a recent study of sorghum, the hexamer “AAAAAA” has been revealed prominently in PACs from both 3’UTR and intron at the same region; and heat, salt and drought stresses do not affect the enrichment [17]. These results indicate that the heat stress could induce the usage of specific sequence signals to confer unique APA-mediated transcripts. But the mechanism of the specificity requires further information from functional analysis of proteins involved in the polyA site selection.

## Methods

### Plant materials and growth conditions

The *P. trichocarpa* Nisqually-1 clone was maintained in a greenhouse as described [29]. For temperature treatments, the aseptic cuttings of *P. trichocharpa* (8–10 cm) were kept in the rooting media [30] for about 30 days before the treatments. The growth chamber was under long-day conditions (16-h light/8-h dark) at 24 °C and 40% humidity. To perform the low temperature treatment, a freezer was controlled by a temperature sensor (PURUI G6000, Ningbo, China). To perform the high temperature treatment, an incubator was set to appropriate temperature prior to the experiment to stabilize the internal temperature. Whole seedlings of at least three individuals were collected for sample preparation.

### PAS-seq library construction, sequencing and data processing

For the sequencing library construction, total RNA was treated with RQ1 DNase (Promega, Madison, USA) to remove DNA. The quality and quantity of the purified RNA were determined by measuring the absorbance at 260 nm/280 nm (A260/A280) using smartspec plus (BioRad, Munich, Germany). RNA integrity was further verified by 1.5% agarose gel electrophoresis. For each sample, 5 μg of total RNA was used for PAS-seq library preparation using SMART RT system. In brief, polyadenylated mRNAs were purified with oligo (dT)-conjugated magnetic beads (Invitrogen, USA). Purified RNA was fragmented, and reverse transcription was performed with a modified RT primer harboring dT18 and two additional anchor nucleotides at the 3′ terminus. Then DNA was synthesized with Terminal-Tagging oligo DNA using ScriptSeq™ v2 RNA-Seq Library Preparation Kit (Illumina, USA). The cDNAs were purified and amplified, and PCR products corresponding to 300–500 bp were purified, quantified and stored at − 80 °C before sequencing. For high-throughput sequencing, the libraries were prepared following the manufacturer’s instructions and applied to Illunima HiSeq X Ten system for 150 nt paired-end sequencing. The reads were filtered for quality checking, and only the end 1 sequences of clean reads were used for downstream analyses. The sequencing and initial reads processing were performed by ABlife Inc. (Wuhan, Hubei Province, China). All sequencing data were deposited under National Center for Biotechnology Information Bioproject accession PRJNA61765.

### Sequence mapping and PAC identification

The reference genome of *Populus trichocarpa* (version 3.1) was downloaded from Phytozome [31]; https://phytozome.jgi.doe.gov). The reads mapping was performed by TopHat2 allowing 2 mismatches [32]. To obtain the expression abundance, the RPKM (Reads per kilobase of a gene per million reads) value was calculated [33].

For identification of polyA-site cluster (PAC), the 3′ mapped polyA reads were initially determined as polyA sites; and then quantified based on the Tag Per Million (TPM) method [TPM (PAC) = reads mapped to polyA site (PAC)*1,000,000/total reads]. The identification of PAC was performed using the CAGEr package [34]; in brief, the polyA sites within 20 nt with TPM over 0.5 were clustered. The PAC within 100 nt in different PAS-Seq libraries were further clustered to generate the PAC sequences (Supple. Dataset 1). For APA analysis, PAC sequences with only one polyA site or TPM less than 3 were filtered. The total PAC sites were independently determined through combing the sequencing reads with TPM cutoff of 5. To identify genes with shifted PACs, the difference of PAC locations was calculated by CAGEr to obtain shift score (Supple. Dataset 2, 3). The Kolmogorov-Smirnov test was performed to identify significant shifts of PACs with *p*-value < 0.01 (Supple. Dataset 2, 3).

### Nucleotide composition and sequence signature analysis

The abovementioned PACs for each sample were used for motif analysis. And the 50 bp upstream and downstream sequences of each PAS were extracted. For the nucleotide distribution analysis, the composition of each PAS at each position were calculated. And the sequence motifs were analyzed using the SignalSleuth2 [28] with the following options: k = 6 (where k is the length of the motif) and top 30 motifs.

For stress-induced analysis, sequences of genes with shifted PACs were extracted according to the position information (Supple. Dataset 4, 5) using an in-house python script. The PACs for each treatment were divided into two groups: one was the PACs under control condition, and another one under the treatment condition. The sequence motif analysis was performed using SignalSleuth2 as mentioned above, and only the PACs shift distance over 50 were used for the analysis.

### Functional enrichment analysis

For functional analysis of shifted genes, the annotation information from Gene Ontology and Kyoto Encyclopedia of Genes and Genomes of *P. trichocarpa* genes were obtained. The enrichment of GO terms and KEGG pathway was identified based on the significance of the hypergeometric tests, and further corrected by FDR (Hochberg). The corrected *p*-values less than 0.05 were determined as significant enrichment. Significantly enriched GO terms were categorized into molecular function, biological process and cellular component as listed in Supple. Dataset 6. For KEGG analysis, the enrichment ratio was calculated as: Enrichment ratio of each KEGG pathway = subset of genes / total number of pathway genes.

## Supplementary Information

**Additional file 1: Table S1.** The overview of PAS-Seq sequencing statistics. CK, control condition; HT, 2 heat stress; LT, cold stress. UniqTag, unique tags of un-redundant reads. DUP, duplication level 3 calculated as the ratio of duplicated reads. **Table S2.** The statistics of reads mapping to the reference genome**.** CK, control 5 condition; HT, heat stress; LT, cold stress. Input, the clean reads. End_A indicates the percentage 6 and number of reads after trimming the As at the end of reads, and the reads are further trimmed at 7 the start of reads (Start_A). End uniq mapped, the number and mapping rate of End_A reads to the 8 reference genome. Start uniq mapped, the number and mapping rate of Start_A reads to the reference 9 genome. Final PAS-reads, the reads after trimming of As for downstream analysis. **Table S3.** Distribution of number and percentages of polyA-site cluster (PAC). CK, 11 control condition; HT, heat stress; LT, cold stress. For combined analysis, the PACs were filtered 12 with TPM > = 5. For individual sample, the PACs were using threshold of TPM > = 3.

**Additional file 2: Dataset 1.** The 50 bp upstream and downstream sequences of PACs detected under control, 15 cold and heat conditions.

**Additional file 3: Dataset 2.** Identification of shifted PACs under heat stress.

**Additional file 4: Dataset 3.** Identification of shifted PACs under cold stress.

**Additional file 5: Dataset 4.** The 50 bp upstream and downstream sequences of PACs of shifted genes under heat stress.

**Additional file 6: Dataset 5.** The 50 bp upstream and downstream sequences of PACs of shifted genes under cold stress.

**Additional file 7: Dataset 6.** The enriched GO terms under heat and cold stresses.

## Data Availability

All data of this work are available from links of the manuscript. The raw sequencing data are available from National Center for Biotechnology Information under Bioproject accession number PRJNA61765.
