# Supplementary material for: Alternative Polyadenylation in response to temperature stress contributes to gene regulation in Populus trichocarpa
Source: BMC Genomics. 2021 Jan 14;22:53. doi: 10.1186/s12864-020-07353-9 (PMC7809742; doi:10.1186/s12864-020-07353-9)
Supplement: Supplementary file 1 — Additional file 1: Table S1. The overview of PAS-Seq sequencing statistics. CK, control condition; HT, 2 heat stress; LT, cold stress. UniqTag, unique tags of un-redundant reads. DUP, duplication level 3 calculated as the ratio of duplicated reads. Table S2. The statistics of reads mapping to the reference genome. CK, control 5 condition; HT, heat stress; LT, cold stress. Input, the clean reads. End_A indicates the percentage 6 and number of reads after trimming the As at the end of reads, and the reads are further trimmed at 7 the start of reads (Start_A). End uniq mapped, the number and mapping rate of End_A reads to the 8 reference genome. Start uniq mapped, the number and mapping rate of Start_A reads to the reference 9 genome. Final PAS-reads, the reads after trimming of As for downstream analysis. Table S3. Distribution of number and percentages of polyA-site cluster (PAC). CK, 11 control condition; HT, heat stress; LT, cold stress. For combined analysis, the PACs were filtered 12 with TPM > = 5. For individual sample, the PACs were using threshold of TPM > = 3. [file 12864_2020_7353_MOESM1_ESM.pdf]

Supplementary information

Supple. Table S1. The overview of PAS-Seq sequencing statistics. CK, control condition; HT, heat stress; LT, cold stress. UniqTag, unique tags of un-redundant reads. DUP, duplication level calculated as the ratio of duplicated reads.

Supple. Table S2. The statistics of reads mapping to the reference genome. CK, control condition; HT, heat stress; LT, cold stress. Input, the clean reads. End_A indicates the percentage and number of reads after trimming the As at the end of reads, and the reads are further trimmed at the start of reads (Start_A). End uniq mapped, the number and mapping rate of End_A reads to the reference genome. Start uniq mapped, the number and mapping rate of Start_A reads to the reference genome. Final PAS-reads, the reads after trimming of As for downstream analysis.

Supple. Table S3. Distribution of number and percentages of polyA-site cluster (PAC). CK, control condition; HT, heat stress; LT, cold stress. For combined analysis, the PACs were filtered with TPM >= 5. For individual sample, the PACs were using threshold of TPM >= 3.

Supple. Dataset 1. The 50 bp upstream and downstream sequences of PACs detected under control, cold and heat conditions.

Supple. Dataset 2. Identification of shifted PACs under heat stress.

Supple. Dataset 3. Identification of shifted PACs under cold stress.

Supple. Dataset 4. The 50 bp upstream and downstream sequences of PACs of shifted genes under heat stress.

Supple. Dataset 5. The 50 bp upstream and downstream sequences of PACs of shifted genes under cold stress.

Supple. Dataset 6. The enriched GO terms under heat and cold stresses.

Supple. Table S1. The overview of PAS-Seq sequencing statistics. CK, control condition; HT, heat stress; LT, cold stress. UniqTag, unique tags of un-redundant reads. DUP, duplication level calculated as the ratio of duplicated reads.

sample	raw	clean	clean_per	uniqTag	raw_base	clean_base	base_per	Q20	Q30	GC	DUP
**CK**	38442134	24439127	63.57%	14589703 (59.7%)	5.77G	3.07G	53.31%	89.98%	80.31%	32%	78.30%
**HT**	36689742	23386957	63.74%	13026085 (55.7%)	5.50G	3.07G	55.85%	90.12%	80.46%	31%	82.26%
**LT**	42983412	27069412	62.98%	15983355 (59.05%)	6.45G	3.49G	54.13%	89.83%	79.89%	33%	78.77%

Supple. Table S2. The statistics of reads mapping to the reference genome. CK, control condition; HT, heat stress; LT, cold stress. Input, the clean reads. End_A indicates the percentage and number of reads after trimming the As at the end of reads, and the reads are further trimmed at the start of reads (Start_A). End uniq mapped, the number and mapping rate of End_A reads to the reference genome. Start uniq mapped, the number and mapping rate of Start_A reads to the reference genome. Final PAS-reads, the reads after trimming of As for downstream analysis.

Sample	Input	End_A	Start_A	End uniq mapped	Start uniq mapped	total merged uniq mapped	Final PAS-reads
**CK**	24439127	9945011 (40.69%)	436622 (1.79%)	5325224 (53.55%)	183495 (42.03%)	5484086 (55.14%)	4902037 (49.29%)
**HT**	23386957	8935917 (38.21%)	390980 (1.67%)	4370962 (48.91%)	148518 (37.99%)	4500543 (50.36%)	3909828 (43.75%)
**LT**	27069412	10187140 (37.63%)	427362 (1.58%)	5261676 (51.65%)	177236 (41.47%)	5415587 (53.16%)	4824585 (47.36%)

Supple. Table S3. Distribution of number and percentages of polyA-site cluster (PAC). CK, control condition; HT, heat stress; LT, cold stress. For combined analysis, the PACs were filtered with TPM >= 5. For individual sample, the PACs were using threshold of TPM >= 3.

Sample	tts_down_1000	5'UTR	3'UTR	CDS	Introns	Intergenic	Antisense
CK	4445 (11.18%)	243 (0.61%)	27568 (69.36%)	1011 (2.54%)	2033 (5.11%)	3606 (9.07%)	842 (2.12%)
HT	4314 (11.49%)	260 (0.69%)	24176 (64.38%)	978 (2.60%)	2643 (7.04%)	3640 (9.69%)	1543 (4.11%)
LT	4614 (11.69%)	207 (0.52%)	27212 (68.92%)	901 (2.28%)	2065 (5.23%)	3593 (9.10%)	889 (2.25%)
Combined analysis	3180 (12.68%)	304 (1.21%)	15631 (62.34%)	860 (3.43%)	1754 (7.00%)	1813 (7.23%)	1533 (6.11%)
